# PAIN INTENSITY AND PAIN CATASTROPHIZING ARE RELATED TO PHYSICAL PERFORMANCE IN OLDER ADULTS WITH BACK PAIN

**DOI:** 10.1093/geroni/igad104.2536

**Published:** 2023-12-21

**Authors:** Corey Simon, Patrick Knox, Adam Goode, Trevor Lentz, Steven George, Gregory Hicks, Cathleen Colón-Emeric, Francis Keefe

**Affiliations:** Duke University, Durham, North Carolina, United States; University of Delaware, Newark, Delaware, United States; Duke University, Durham, North Carolina, United States; Duke University, Durham, North Carolina, United States; Duke University, Durham, North Carolina, United States; University of Delaware, Newark, Delaware, United States; Duke University School of Medicine, Durham, North Carolina, United States; Duke University, Durham, North Carolina, United States

## Abstract

Back pain is highly prevalent in older adults and a risk factor for poor/declining physical performance. Back pain intensity and pain catastrophizing are two factors known to be associated with physical performance in younger adults; though a paucity of similar research exists in older adults. Thirty-nine older adults with back pain completed a battery of four standardized physical performance tests: 30-second chair rise, repetitive seated trunk rotation, repetitive standing functional reaching, and six-minute walk. Test scores were z-transformed and summated for a standardized physical performance score. We measured resting pain intensity and dispositional pain catastrophizing before the battery and movement-evoked pain intensity and situational pain catastrophizing during/after the battery. After accounting for age and biological sex, resting and movement-evoked pain intensity explained 14% and 39% unique variance in physical performance. In multivariable models, movement-evoked pain was the stronger factor. Similar modeling showed dispositional and situational pain catastrophizing explained 14% and 28% unique variance in physical performance. In multivariable models, situational pain catastrophizing was the stronger factor. Finally, path modeling showed situational pain catastrophizing mediated the total effect of movement-evoked pain on physical performance (21%). Findings suggest: 1) Factors with movement (i.e., movement-evoked pain, situational pain catastrophizing) are more strongly associated with physical performance than factors measured independent of movement (i.e., resting pain, dispositional pain catastrophizing); and 2) The impact of pain on physical performance is mediated by psychological distress. Importantly, neither movement-evoked pain nor situational pain catastrophizing are part of current standard of care for older adults with back pain.

